# 

**Published:** 1875-08

**Authors:** 


**TERMS, ALWAYS IN ADVANCE:** One copy, per annum.... $4 00

☞ Yearly subscriptions may begin with any number, Postage Free.

☞ Persons who send subscriptions for the JOURNAL will please consider the first number received after subscription as a sufficient receipt for money sent.

☞ We cannot return rejected manuscripts.

